# New Drugs, Appliances, and Things Medical

**Published:** 1892-06-18

**Authors:** 


### NEW DRUGS, APPLIANCES, AND THINGS MEDICAL.

[All preparations, appliances, novelties, etc., of which a notice is desired, should be sent for The Editor, to care of The Manager, 140, Strand, London, W.C.]

### MARRIOTT'S PATENT LENTILINE BISCUITS.

(E. Marriott and Co., Hastings.)

We have received a sample of the above for examination and notice. After careful investigation we find them to be well manufactured, pleasant to the taste, free from impurity, and of very considerable nutritive value. From early ages lentils have been one of the stock bean foods of the East. By some it is conjectured the "mess of red pottage" was a form of lentil soup. Like all beans lentils require very careful preparation before eating. In the above biscuits the objections to the crude form of lentil flour have been quite done away with, the result being a distinct and pleasing novelty in biscuits, which are suitable both for invalids and the robust.

### NIEMANN'S CARNINE SYRUP.

(J. H. Niemann and Co., and R. F. Maclorky, 12, Major Street, Manchester.)

Since it has been proved that meat extracts are a stimulant only, and that uncooked meat juice is the most valuable invalid food, we have examined many specimens of the the latter. As a rule the juice has to be preserved by some chemical. It has been reserved to the above company to use pure cane sugar as the preservative. The result is a delicately-flavoured meat syrup, which we are sure will find favour, not only as a valuable food and stimulant, but also as a change from other forms of meat juice. We have examined the preparation with considerable interest. It is a light claret or carmine-coloured syrup of pleasant appearance, and when diluted, of equally pleasant flavour. On heating in a test-tube, a really solid coagulum results. This indicates a very high percentage of soluble albumen, and as this soluble albumen is what is wanted in the feeding of the sick, the inference is that the syrup is an excellent invalid food. The syrup may be taken diluted with ærated water, with a small amount of lemon juice (excess coagulates it) to qualify the sweetness, in cold or lukewarm water (heat also coagulates it), or in cocoa, but not with tea or coffee, as the tannin present forms an insoluble tannate of albumen with the syrup. We have to thank the inventors for yet another aid to the troublesome task of feeding the sick.

### PETERMAN'S COCKROACH AND BEETLE FOOD.

(J. F. Shorey, 67, Farringdon Road.)

This well-known non-poisonous preparation has maintained its reputation fully for the past two years. We noticed it as being by far the best preparation of the kind known to us, and we can speak from personal experience of the benefits derived from its use. We note that most of the large hotels, the P. and O. Company, several of the leading clubs, and large establishments like Whiteley's, use it largely.

